# Uniportal thoracoscopic mediastinal lymph node dissection: Technical pearls and anatomical considerations

**DOI:** 10.1016/j.xjtc.2025.10.009

**Published:** 2025-11-11

**Authors:** Tong Li, Yang Zhang

**Affiliations:** a Department of Thoracic Surgery and State Key Laboratory of Genetics and Development of Complex Phenotypes, Fudan University Shanghai Cancer Center, Shanghai, China; b Institute of Thoracic Oncology, Fudan University, Shanghai, China; c Department of Oncology, Shanghai Medical College, Fudan University, Shanghai, China

**Figure:**
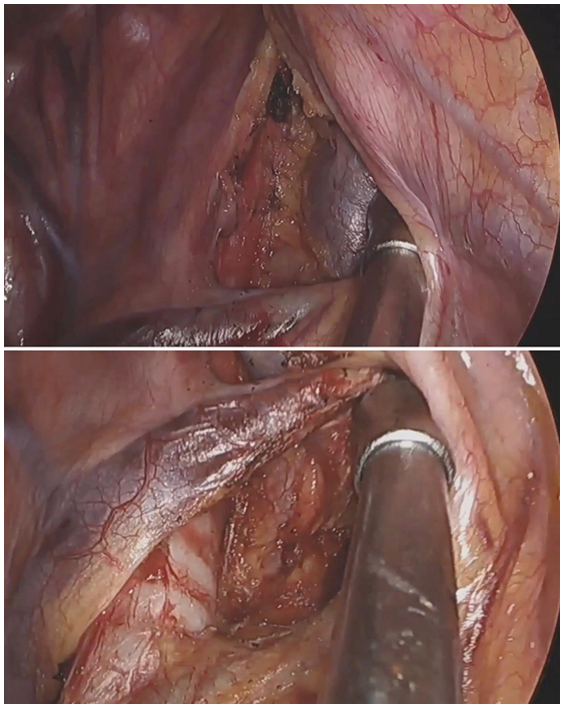
Anatomic landmarks for uniportal VATS dissection of stations 2R and 4R.

**Central Message**

Uniportal VATS achieves complete MLND through standardized, station-specific, nerve-sparing techniques, preserving minimal invasiveness while maintaining oncologic adequacy.

The shift from multiportal to uniportal video-assisted thoracoscopic surgery (VATS) has reduced postoperative pain and accelerated recovery.^1^ Yet the single-incision approach narrows working angles and limits instrument triangulation, making mediastinal lymph node dissection (MLND) technically demanding.^2^ A key goal in MLND is the en bloc removal of all lymph nodes and surrounding fatty tissue within the clear anatomic boundaries of each station, following International Association for the Study of Lung Cancer guidelines.^3^ This is important for both accurate staging and oncological outcomes. In our practice, we emphasize a complete anatomic zone-based dissection to ensure a thorough resection. Detailed, station-specific techniques, particularly accounting for anatomic variation, remain sparsely described.^4^, ^5^, ^6^, ^7^, ^8^ We provide a step-by-step, station-based guide to complete MLND via uniportal VATS, grounded in consistent anatomic landmarks and practical maneuvers to overcome uniportal constraints.

## Technical Pearls and Key Steps

### Considerations for Patient Selection

Although the oncological indications for uniportal MLND mirror those for the multiportal approach, careful patient selection is crucial. We found that the uniportal technique is less suitable for cases where optimal exposure is challenging, such as in the presence of extensive pleural adhesions or bulky nodal disease. In such situations, we have a low threshold for converting to multiportal or open techniques to prioritize the principles of safe and complete resection.

### Instrument Handling and Traction Techniques

In our uniportal VATS technique, the way we handle instruments is very important. The entire procedure was performed using a harmonic scalpel in conjunction with a standard Yankauer suction device, which effectively minimized smoke production, reduced bleeding, and maintained a clear surgical field throughout the MLND. A key part of our approach is the “external crossing” maneuver, where we intentionally cross the suction and the harmonic scalpel outside the chest. This simple trick helps us position the tips of the tools more accurately inside, making it easier to reach and dissect tissues. During dissection—especially when using the “peeling technique” for lymph nodes—the suction is used to provide gentle traction, while the curved tip of the harmonic scalpel carefully separates the node from nearby structures. We use small and precise movements to avoid damaging vessels or nerves. It is important not to pull too hard and to always follow the natural tissue planes. Because we work through only 1 port, we sometimes switch the suction and scalpel between our hands. Being comfortable using both hands with both instruments is essential for smooth and safe surgery. Good communication with the camera assistant is also needed to maintain a stable view throughout the procedure.

### Right Side

Position the patient in left lateral decubitus with approximately 30° right-anterior tilt. Create a 3- to 4-cm utility incision in the fourth or fifth intercostal space along the anterior axillary line. All steps can be performed with an ultrasonic scalpel as the sole energy device, as demonstrated in Videos 1-5.

### Stations 2R and 4R

**Figure 1:**
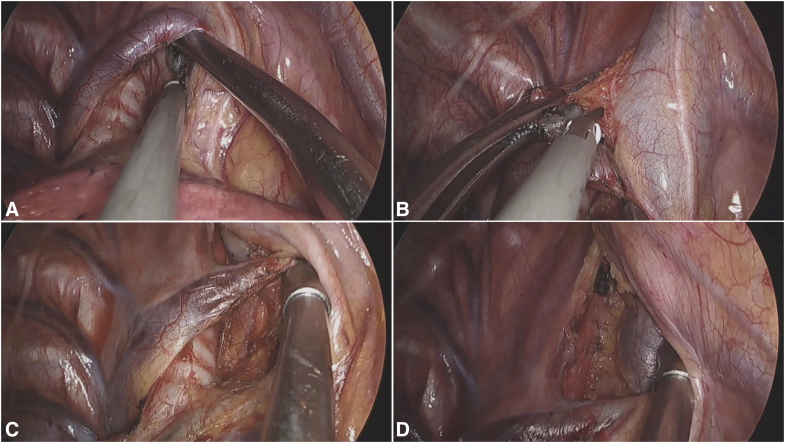
Key anatomic relationships and surgical steps for MLND of stations 2R/4R. A, Initial exposure of the “triangle of safety,” bounded by the SVC, trachea, and azygos vein. B, Schematic illustrating the “peeling technique” used for en bloc dissection of the lymph node chain. C and D, Final surgical field after complete en bloc removal of the nodal tissue, showing the exposed trachea, posterior wall of the SVC, and azygos vein arch.

The continuous lymphatic chain from the thoracic inlet (2R) to the azygos vein (4R) requires meticulous dissection along 3 critical planes: tracheal, venous, and neural. After ventral table tilt (30°), initial exposure begins with identification of the “triangle of safety” bordered by the superior vena cava (SVC), trachea, and azygos vein. The mediastinal pleura is opened along the posterior SVC border using ultrasonic scalpels, with careful preservation of the right vagus nerve. The “peeling technique” involves sequential separation of nodal tissue from the tracheal wall (using upward traction on nodes), SVC posterior wall (with medial retraction), and azygos arch (after cephalad displacement). For superior attachments near the brachiocephalic artery, meticulous dissection with the energy device prevents inadvertent vascular injury. The recurrent laryngeal nerve (RLN) is protected by maintaining dissection anterior to the tracheoesophageal groove and limiting energy use in this region. Complete en bloc removal is achieved by dividing the apical nodal attachments after confirming complete separation from all 3 anatomic planes (Figure 1 and Video 1).

### Stations 7 and 8

**Figure 2:**
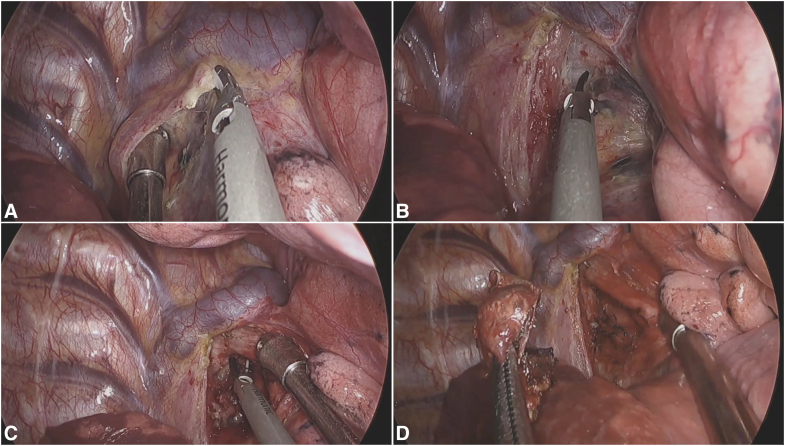
Systematic approach to uniportal MLND of stations 7 and 8. A, Initial exposure and pleural incision along the vagus nerve, extending from the inferior pulmonary vein to the azygos arch. B, Key step 1: releasing adhesions between the inferior pulmonary vein and the esophagus. C, Key step 2: fully exposing the carinal angle to define the superior boundary of the dissection. D, Completion of the en bloc resection. The final field shows the exposed esophagus, pericardium, and carina with the right and left main bronchi, confirming a thorough dissection.

The dissection requires 30° ventral table tilt and lower lobe retraction. The procedure begins by incising the posterior mediastinal pleura along the course of the vagus nerve, extending from the inferior border of the inferior pulmonary vein to the lower margin of the azygos arch. Meticulous dissection first addresses the nodal-esophageal interface. Critical maneuvers include complete release of adhesions between the inferior pulmonary vein and the esophagus, followed by full exposure of the carinal angle. The suction device is then used to displace the nodal package toward the pericardium, facilitating precise separation from the left main bronchus. Titanium clips are recommended for securing significant bronchial arteries. The dissection then proceeds to systematically separate the nodal-pericardial attachments, followed by sequential division of adhesions to the bronchus intermedius, right main bronchus, and finally the carinal residues. This systematic approach ensures complete en bloc resection, which is accomplished primarily through ultrasonic dissection combined with strategic suction traction to maintain proper tissue plane visualization (Figure 2 and Video 2).

### Left Side

Right lateral decubitus position with approximately 30° left-anterior tilt optimizes exposure, with the incision placed in the fourth or fifth intercostal space anterior-axillary line.

### Station 4L

**Figure 3:**
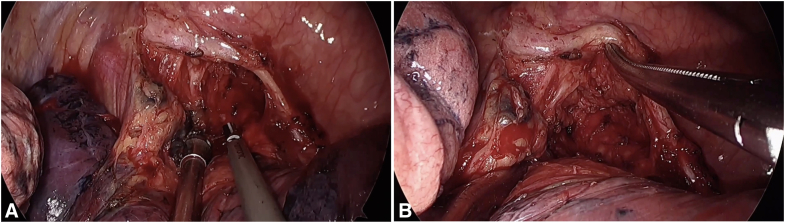
Key steps in station 4L lymph node dissection. A, Optimizing exposure for dissection. The pulmonary artery is depressed anteriorly with the suction tip while superior traction is applied to the nodal package. This maneuver widens the surgical plane between the vessels and the nodal tissue. B, Completing the deep dissection along the trachea. The final view shows the cleared plane along the trachea, with the pulmonary artery defining the anterior boundary. Note that blunt dissection is preferred near the RLN, with energy devices used only in areas clearly distant from the nerve to prevent injury.

The dissection demands precise identification of 3 neural structures: vagus nerve, phrenic nerve, and RLN. After opening the mediastinal pleura between the phrenic and vagus nerves, the RLN is identified, where it loops under the aortic arch near the ligamentum arteriosum. The dissection proceeds from caudal to cephalad along the trachea, using the pulmonary artery as an anterior boundary. A key technical pearl involves using the suction device to depress the pulmonary artery while simultaneously retracting the nodal package superiorly, creating optimal exposure for ultrasonic dissection. The deep plane along the trachea requires careful blunt dissection to avoid RLN injury, with energy application limited to clearly visualized tissue away from neural structures (Figure 3 and Video 3).

### Stations 5 and 6

**Figure 4:**
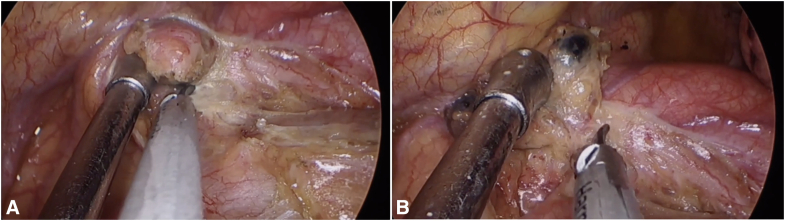
Surgical technique for stations 5 and 6 MLND. A, Incising the mediastinal pleura. The incision is made along the phrenic nerve, from the superior pulmonary vein to the aortic arch, with anterior displacement of the nodal tissue to safeguard the vagus nerve. B, Final dissection with nerve protection. Anterior retraction of the phrenic nerve exposes the nodal tissue, which is then safely dissected and removed en bloc using the “peeling technique.”

The anatomic boundaries of the aortopulmonary window are defined superiorly by the inferior border of the aortic arch, inferiorly by the superior border of the left pulmonary artery, anteriorly by the phrenic nerve, and posteriorly by the vagus nerve. The initial step involves a posterior approach to dissect the mediastinal pleura overlying the vagus nerve. The nodal package is then displaced anteriorly while maintaining the vagus nerve under direct visualization. Anterior mobilization along the lateral edge of the phrenic nerve allows for incision of the mediastinal pleura from the inferior border of the superior pulmonary vein cephalad to the aortic arch, followed by medial mobilization of the nodal tissue toward the phrenic nerve. The “peeling technique” is performed with anterior retraction of the phrenic nerve using a suction device, which facilitates en bloc resection while minimizing the risk of nerve injury (Figure 4 and Video 4).

### Stations 7 and 8

Dissecting lymph nodes at stations 7 and 8 on the left side is particularly challenging due to obstruction from the descending aorta and the close adherence of the left main bronchus. To address these issues, we rely on a stepwise approach that emphasizes exposure and careful instrument handling. The procedure starts by opening the mediastinal pleura along the vagus nerve, extending from below the inferior pulmonary vein up to the carinal angle. We find it helpful to clearly identify these upper and lower landmarks—the carina above and the inferior pulmonary vein below—to establish a broader working space. This principle of “trading width for depth” significantly improves access to deeper structures. To enhance visibility, we retract the lung gently forward and downward, typically using the suction device or a sponge holder.

**Figure 5:**
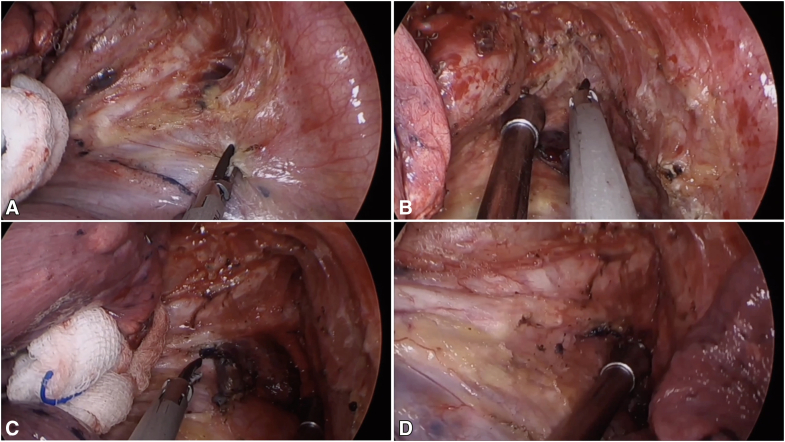
Left-sided dissection of stations 7 and 8 lymph nodes. A, Initial exposure. The mediastinal pleura is opened along the vagus nerve from the inferior pulmonary vein to the carina. Anterior and inferior lung retraction establishes a broad working field, applying the “width for depth” principle. B-D, Key dissection sequence. The procedure proceeds systematically: (B) The nodal tissue is first separated from the esophagus with concurrent exposure of the carinal area; (C) it is then detached from the left main bronchus and the pericardium; and (D) finally, the remaining attachments to the carina are released to complete the en bloc resection.

The dissection itself follows a structured sequence: First, the nodal tissue is separated from the esophagus while progressively exposing the carinal region; second, we ensure clear visualization of the right main bronchus from below; third, adhesions between the nodes and the right bronchus are divided, lifting the tissue toward the carina; then, the nodes are detached from the pericardium and the left main bronchus. This combination of wide exposure and systematic dissection allows for safe and complete removal of stations 7 and 8 lymph nodes (Figure 5 and Video 5).

### Technical Considerations for Challenging Situations

For stations with increased risk of nerve injury (eg, stations 4L, 5, and 6), particular caution is exercised when operating near the recurrent and phrenic nerves. In cases with distorted anatomy or dense adhesions, several specific measures are taken to maximize safety. First, dissection begins in areas where tissue planes are more recognizable, gradually progressing toward the zone of scarring. Traction is applied gently to avoid avulsing small neural branches. In situations where the tissue is particularly fused, we first create a plane using a dissector or suction tip before using any energy device. This allows for the development of a safe working space between the nerve and the surrounding fibrotic tissue. Although intraoperative neuromonitoring is not used routinely, we rely on high-definition magnified visualization to identify and preserve neural structures. The combination of patience, precise exposure, and judicious energy use helps prevent nerve injury.

## Conclusions

Uniportal VATS MLND is feasible but technically demanding, requiring detailed knowledge of mediastinal anatomy and disciplined single-port instrument choreography and energy control. Technological advances—particularly the development of articulating instruments and 3-dimensional visualization systems—are likely to enhance the precision and safety of this procedure. Articulating instruments improve maneuverability behind vascular structures and within narrow spaces such as stations 4L, 7, and 8, reducing instrument conflict and increasing access. Meanwhile, 3-dimensional imaging offers superior depth perception, which facilitates identification of nerves and vessels and helps dissect along tissue planes with greater accuracy. These innovations may not only support more thorough station-by-station dissection and improved nerve preservation but also help shorten the learning curve associated with uniportal MLND. Future studies should aim to standardize the definition and quality assessment of lymph node dissection in uniportal VATS and prospectively evaluate long-term oncological outcomes compared with multiportal approaches.

## Conflict of Interest Statement

The authors reported no conflicts of interest.

The *Journal* policy requires editors and reviewers to disclose conflicts of interest and to decline handling or reviewing manuscripts for which they may have a conflict of interest. The editors and reviewers of this article have no conflicts of interest.

## Supplementary Data

Video 1 Surgical video of MLND at right-sided stations 2R and 4R. Video available at: https://www.jtcvs.org/article/S2666-2507(25)00466-3/fulltext.

Video 2 Surgical video of MLND at right-sided stations 7 and 8. Video available at: https://www.jtcvs.org/article/S2666-2507(25)00466-3/fulltext.

Video 3 Surgical video of MLND at left-sided station 4L. Video available at: https://www.jtcvs.org/article/S2666-2507(25)00466-3/fulltext.

Video 4 Surgical video of MLND at left-sided stations 5 and 6. Video available at: https://www.jtcvs.org/article/S2666-2507(25)00466-3/fulltext.

Video 5 Surgical video of MLND at left-sided stations 7 and 8. Video available at: https://www.jtcvs.org/article/S2666-2507(25)00466-3/fulltext.
