# Supplementary figures and images for: Uniportal thoracoscopic mediastinal lymph node dissection: Technical pearls and anatomical considerations
Source: JTCVS Tech. 2025 Nov 11;35:102133. doi: 10.1016/j.xjtc.2025.10.009 (PMC12881802; doi:10.1016/j.xjtc.2025.10.009)

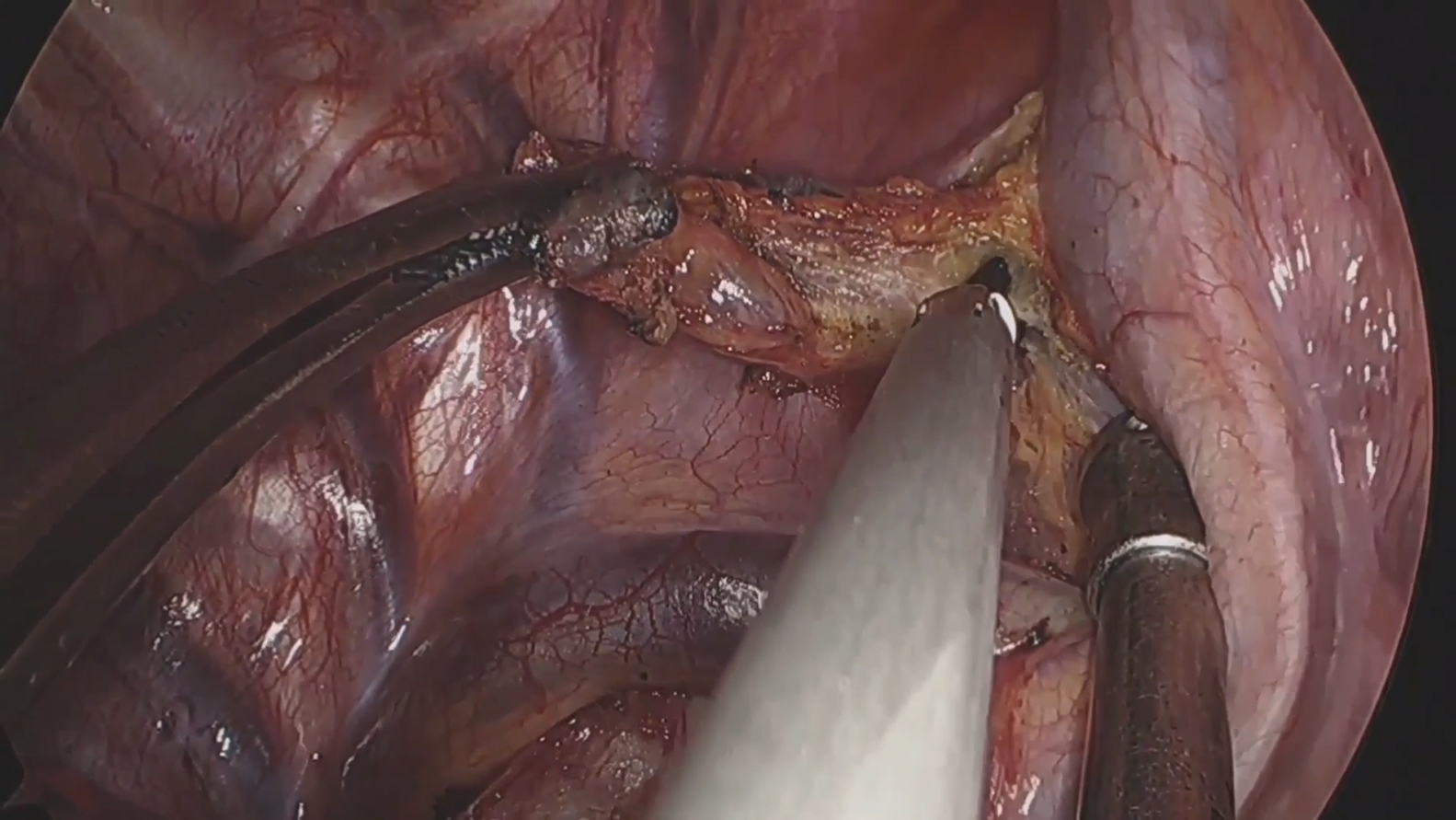

Supplement: Video 1 — Surgical video of MLND at right-sided stations 2R and 4R. Video available at: https://www.jtcvs.org/article/S2666-2507(25)00466-3/fulltext. [file fx2.jpg]

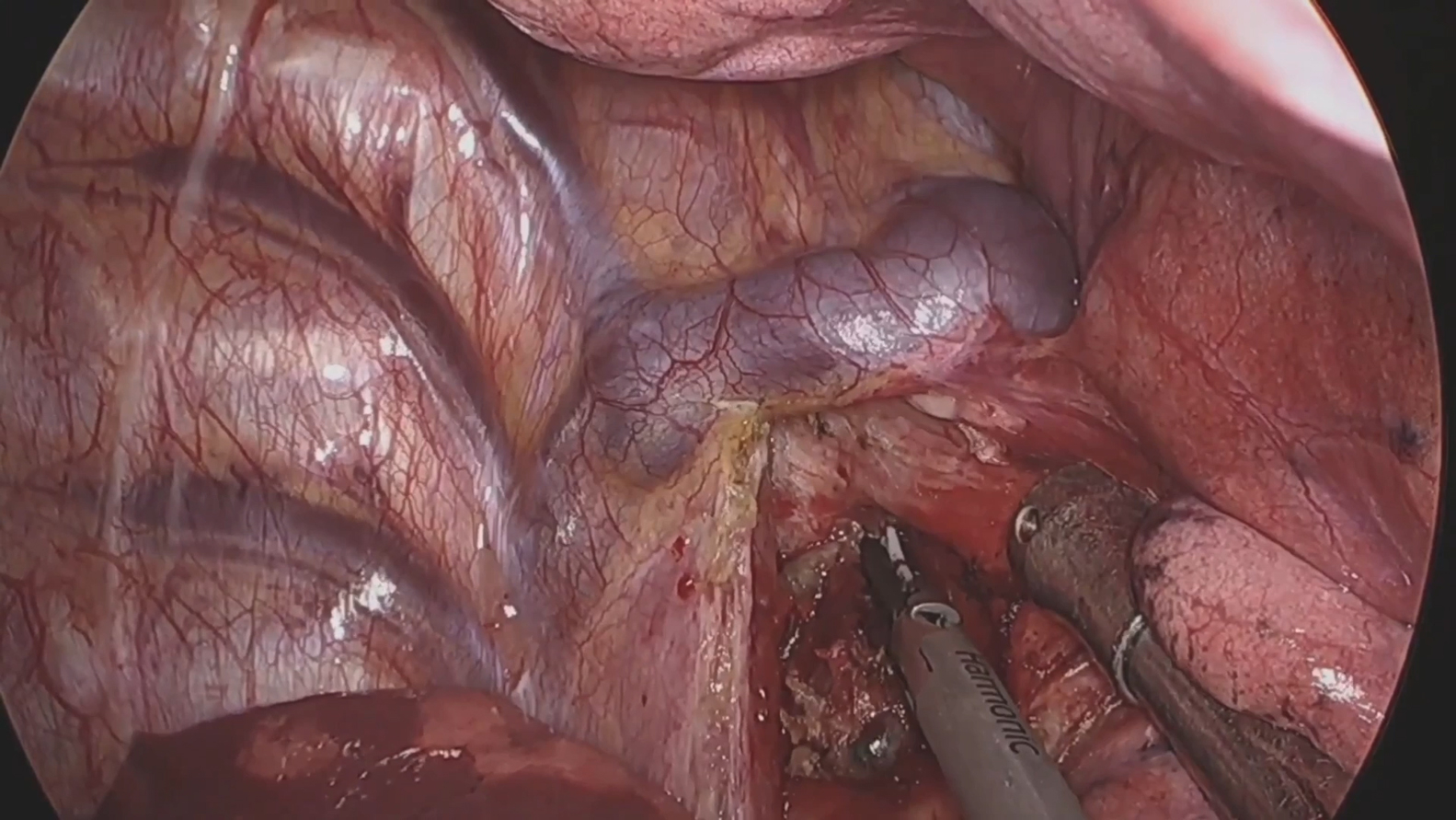

Supplement: Video 2 — Surgical video of MLND at right-sided stations 7 and 8. Video available at: https://www.jtcvs.org/article/S2666-2507(25)00466-3/fulltext. [file fx3.jpg]

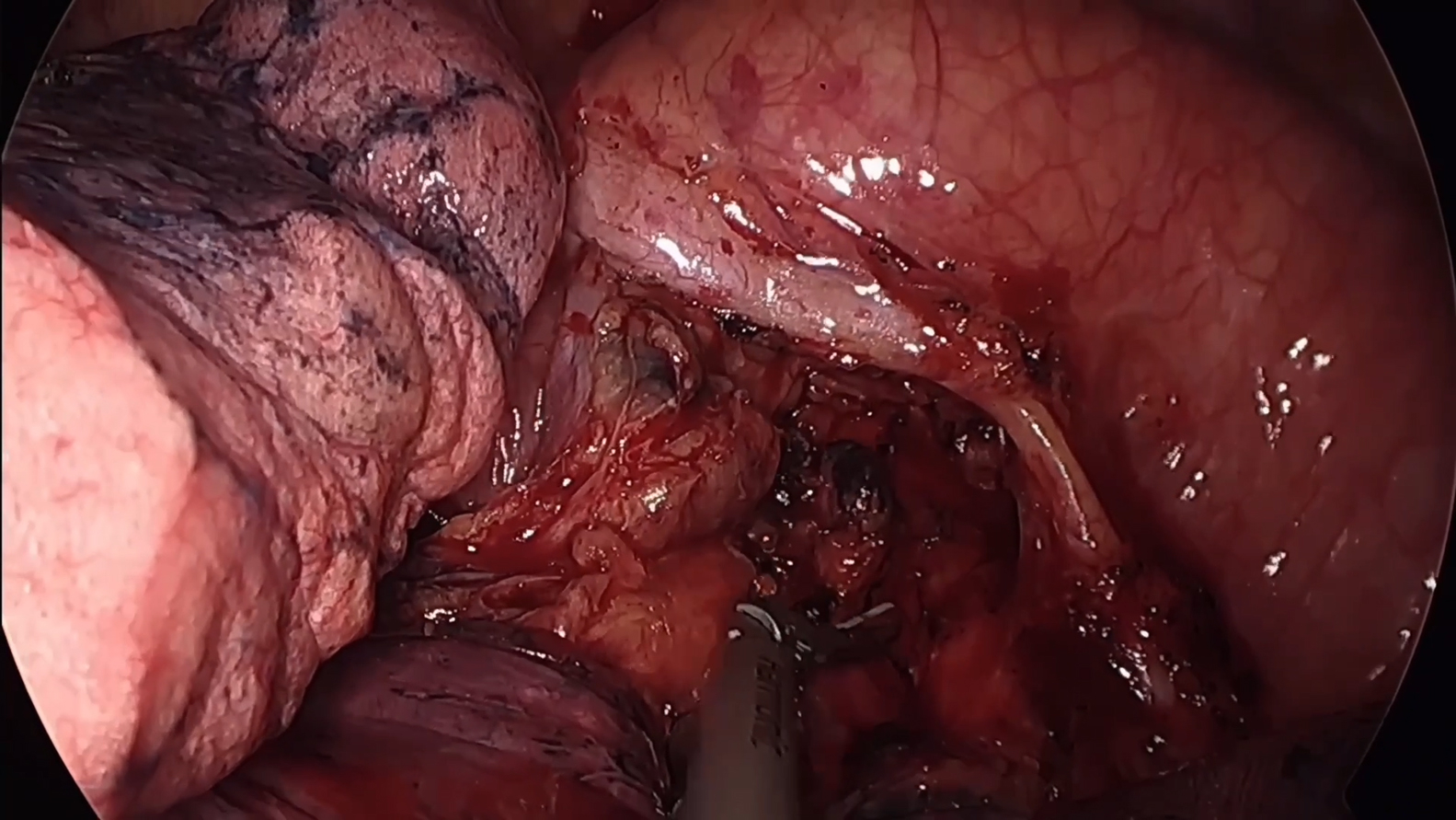

Supplement: Video 3 — Surgical video of MLND at left-sided station 4L. Video available at: https://www.jtcvs.org/article/S2666-2507(25)00466-3/fulltext. [file fx4.jpg]

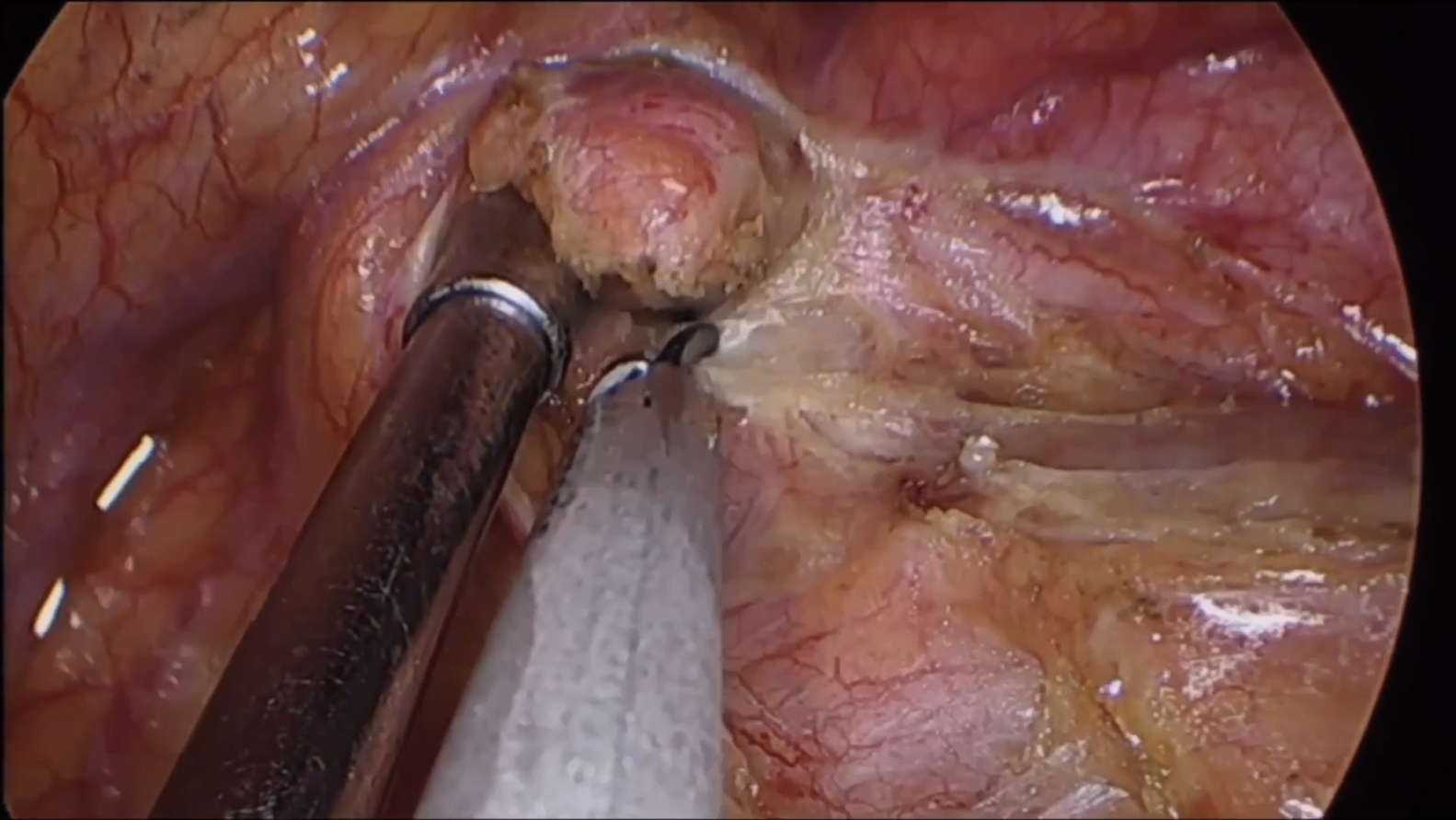

Supplement: Video 4 — Surgical video of MLND at left-sided stations 5 and 6. Video available at: https://www.jtcvs.org/article/S2666-2507(25)00466-3/fulltext. [file fx5.jpg]

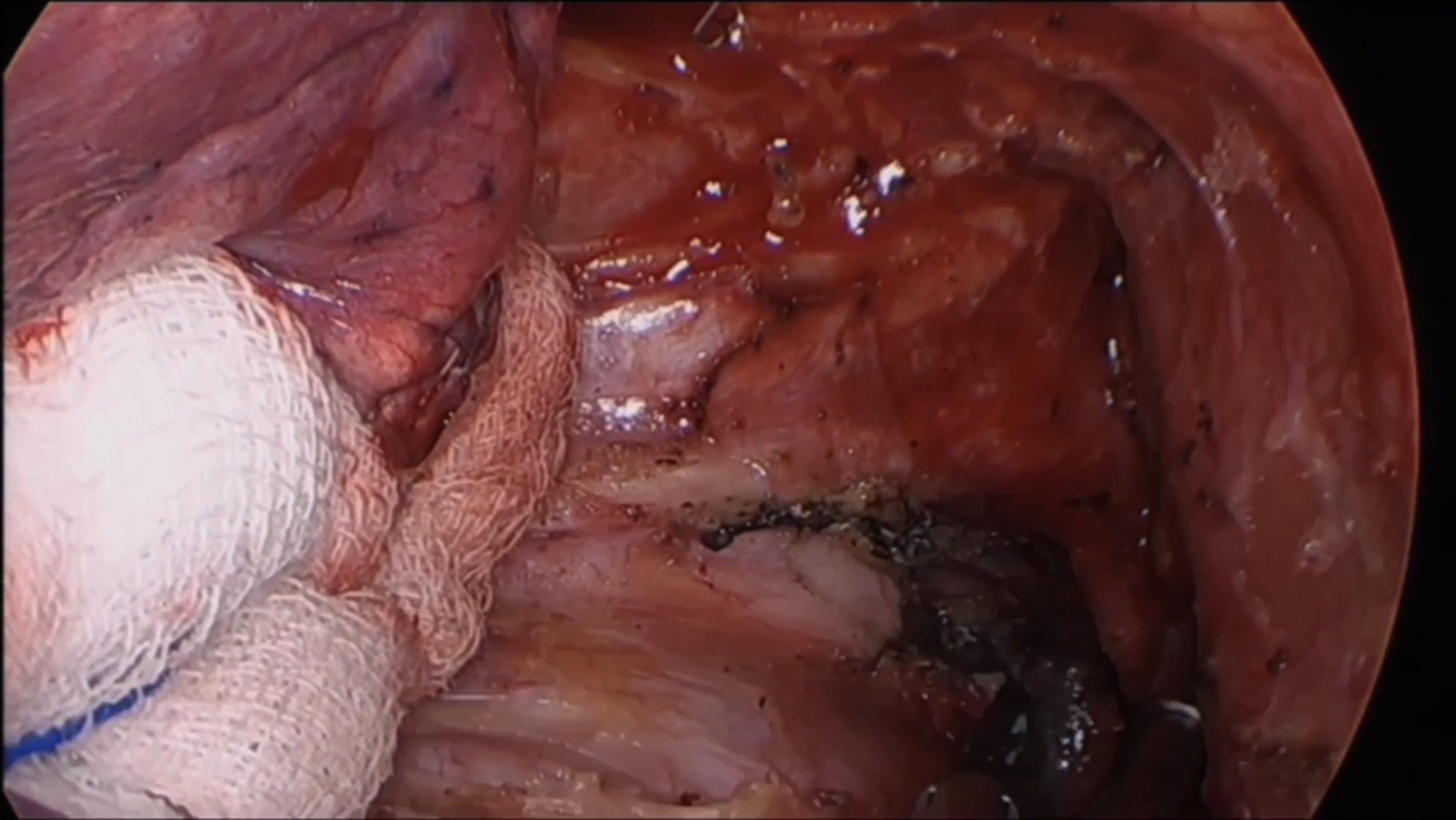

Supplement: Video 5 — Surgical video of MLND at left-sided stations 7 and 8. Video available at: https://www.jtcvs.org/article/S2666-2507(25)00466-3/fulltext. [file fx6.jpg]
